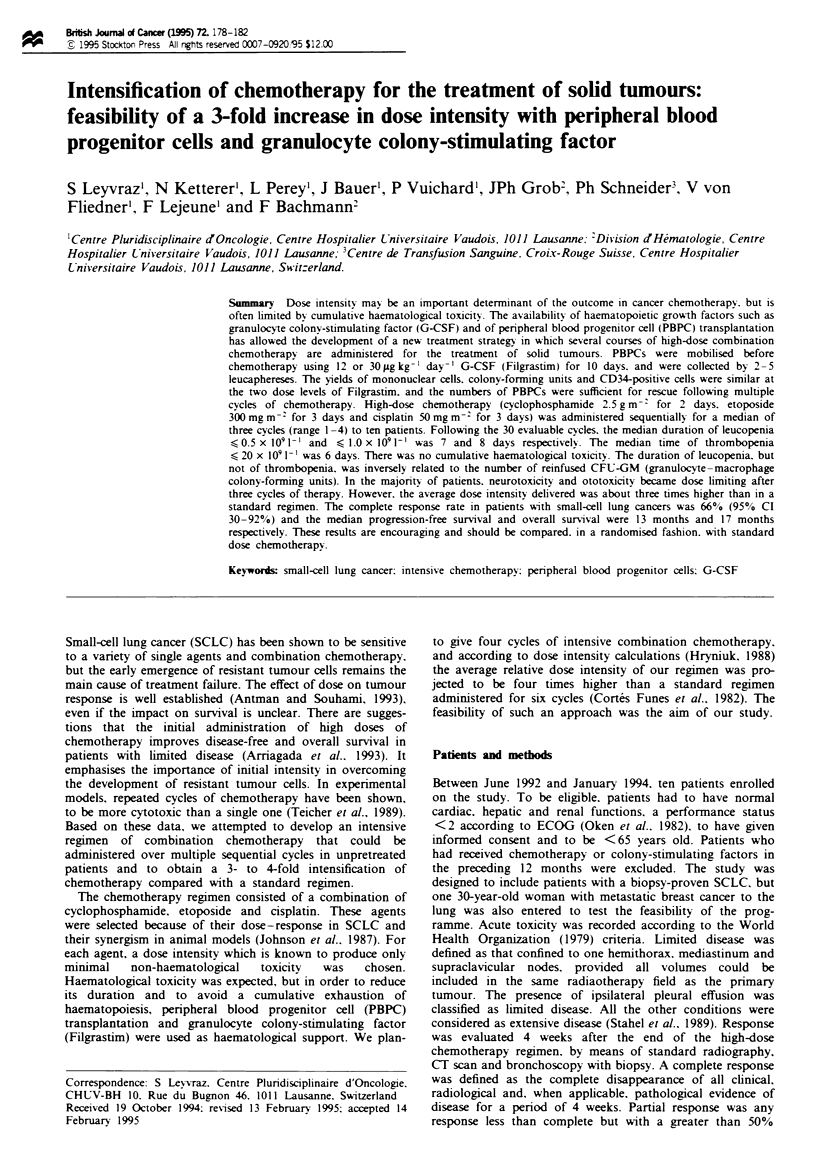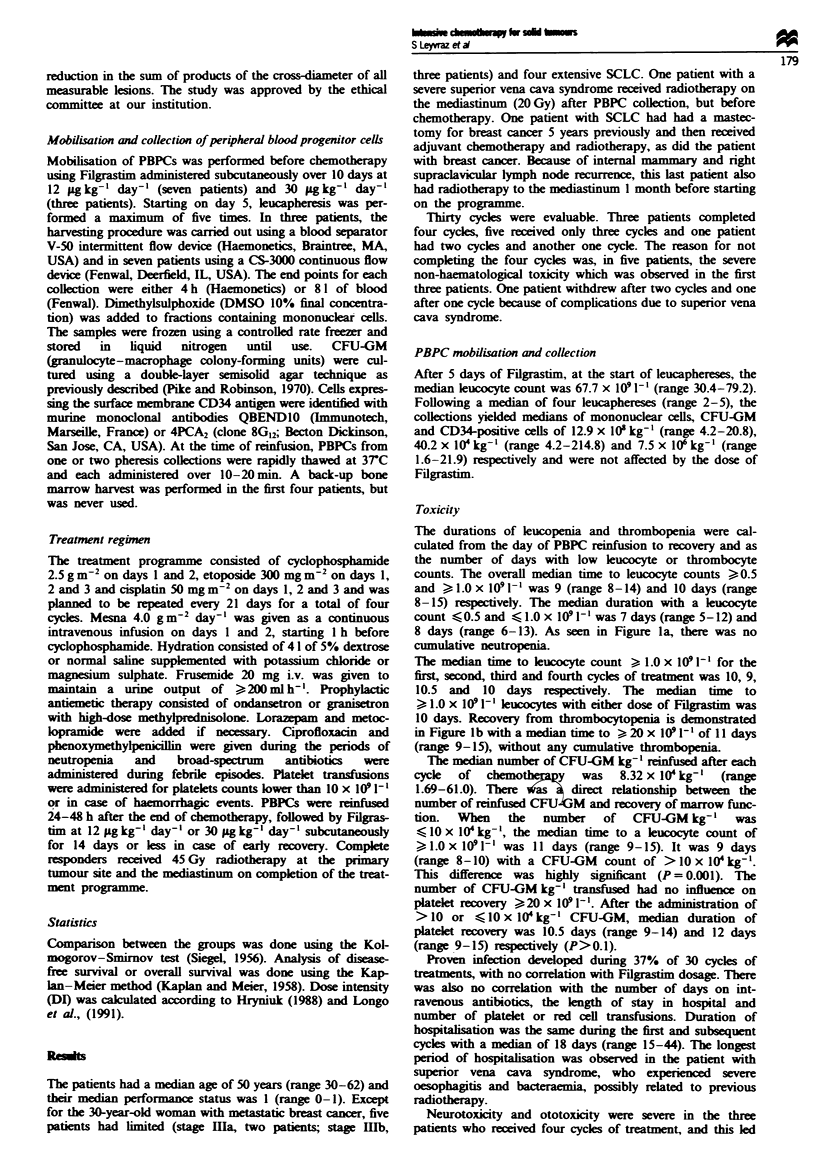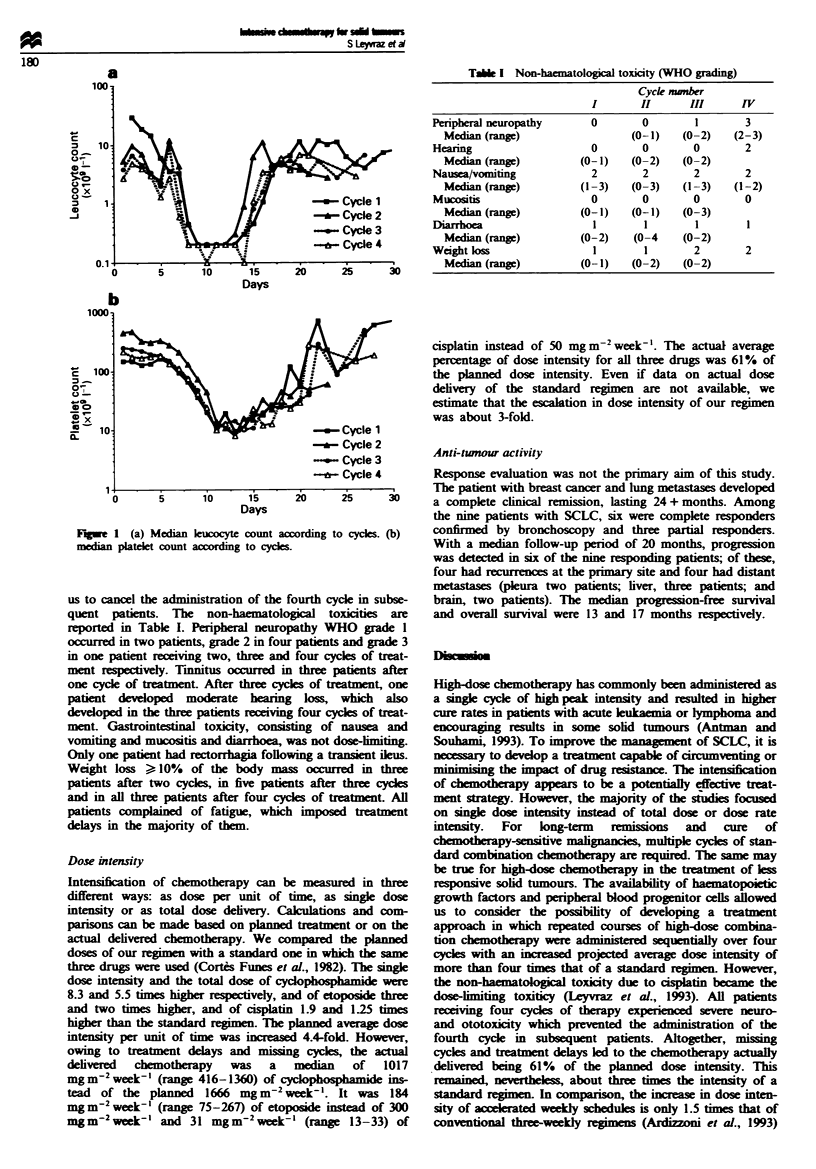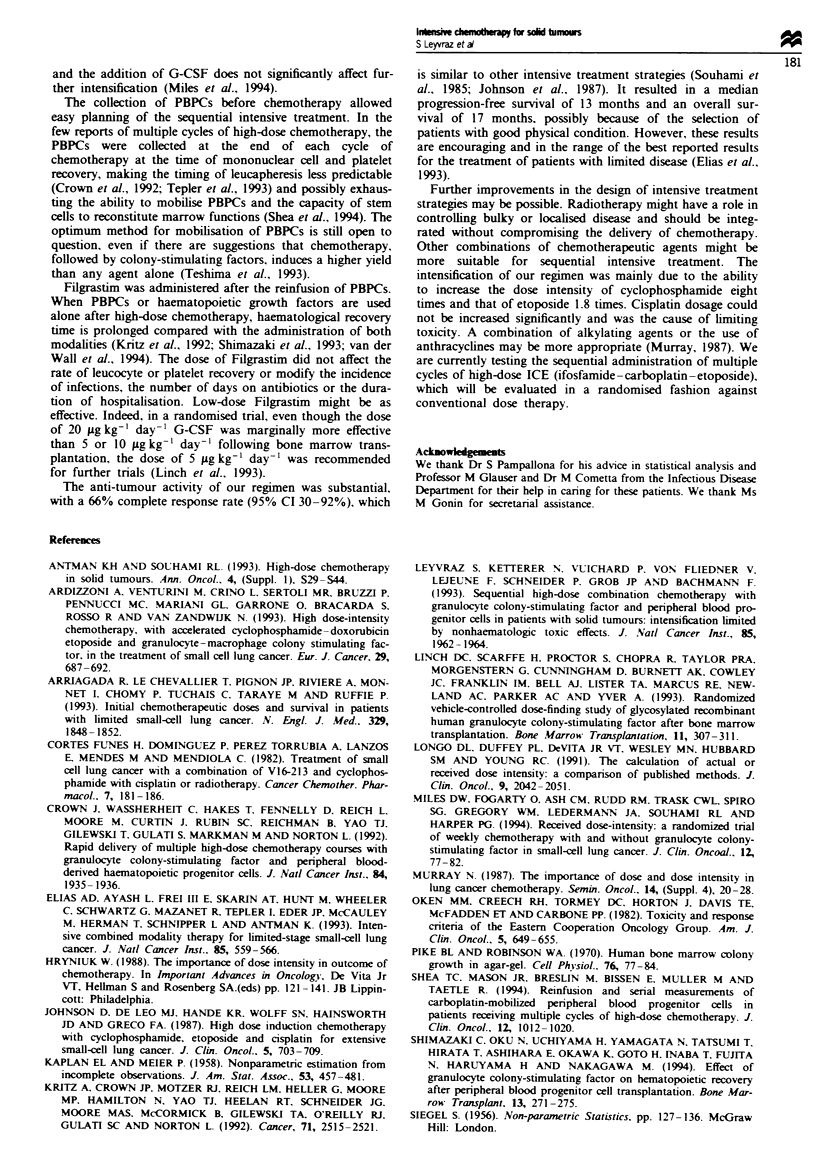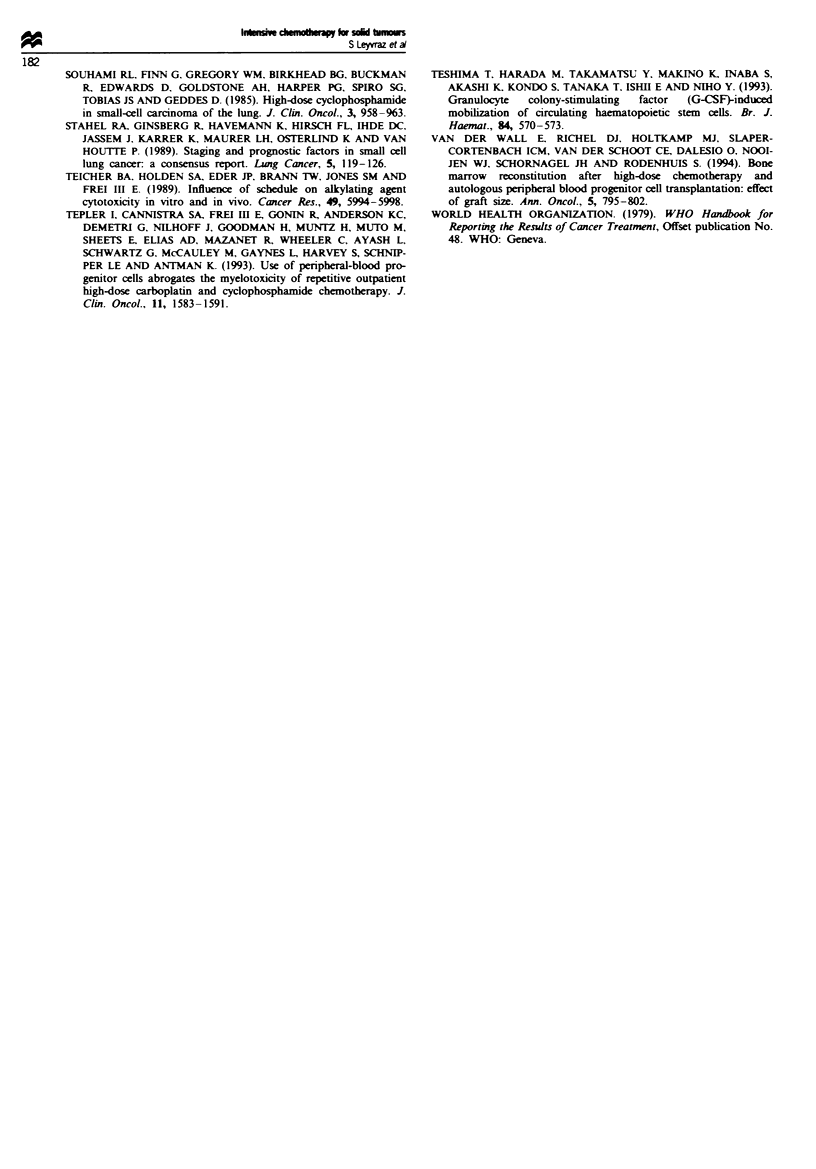# Intensification of chemotherapy for the treatment of solid tumours: feasibility of a 3-fold increase in dose intensity with peripheral blood progenitor cells and granulocyte colony-stimulating factor.

**DOI:** 10.1038/bjc.1995.298

**Published:** 1995-07

**Authors:** S. Leyvraz, N. Ketterer, L. Perey, J. Bauer, P. Vuichard, J. P. Grob, P. Schneider, V. von Fliedner, F. Lejeune, F. Bachmann

**Affiliations:** Centre Pluridisciplinaire d'Oncologie, Centre Hospitalier Universitaire Vaudois, Lausanne, Switzerland.

## Abstract

Dose intensity may be an important determinant of the outcome in cancer chemotherapy, but is often limited by cumulative haematological toxicity. The availability of haematopoietic growth factors such as granulocyte colony-stimulating factor (G-CSF) and of peripheral blood progenitor cell (PBPC) transplantation has allowed the development of a new treatment strategy in which several courses of high-dose combination chemotherapy are administered for the treatment of solid tumours. PBPCs were mobilised before chemotherapy using 12 or 30 micrograms kg-1 day-1 G-CSF (Filgrastim) for 10 days, and were collected by 2-5 leucaphereses. The yields of mononuclear cells, colony-forming units and CD34-positive cells were similar at the two dose levels of Filgrastim, and the numbers of PBPCs were sufficient for rescue following multiple cycles of chemotherapy. High-dose chemotherapy (cyclophosphamide 2.5 g m-2 for 2 days, etoposide 300 mg m-2 for 3 days and cisplatin 50 mg m-2 for 3 days) was administered sequentially for a median of three cycles (range 1-4) to ten patients. Following the 30 evaluable cycles, the median duration of leucopenia < or = 0.5 x 10(9) l-1 and < or = 1.0 x 10(9) l-1 was 7 and 8 days respectively. The median time of thrombopenia < or = 20 x 10(9) l-1 was 6 days. There was no cumulative haematological toxicity. The duration of leucopenia, but not of thrombopenia, was inversely related to the number of reinfused CFU-GM (granulocyte-macrophage colony-forming units). In the majority of patients, neurotoxicity and ototoxicity became dose limiting after three cycles of therapy. However, the average dose intensity delivered was about three times higher than in a standard regimen. The complete response rate in patients with small-cell lung cancers was 66% (95% CI 30-92%) and the median progression-free survival and overall survival were 13 months and 17 months respectively. These results are encouraging and should be compared, in a randomised fashion, with standard dose chemotherapy.


British Journal of Cancer (1995) 72, 178-182

© 1995 Stockton Press All rights reserved 0007-0920/95 $12.00

### Intensification of chemotherapy for the treatment of solid tumours: feasibility of a 3-fold increase in dose intensity with peripheral blood progenitor cells and granulocyte colony-stimulating factor

S Leyvraz[1], N Ketterer[1], L Perey[1], J Bauer[1], P Vuichard[1], JPh Grob[2], Ph Schneider[3], V von Fliedner[1], F Lejeune[1] and F Bachmann[2]

[1]*Centre Pluridisciplinaire d'Oncologie, Centre Hospitalier Universitaire Vaudois, 1011 Lausanne;* [2]*Division d'Hématologie, Centre Hospitalier Universitaire Vaudois, 1011 Lausanne;* [3]*Centre de Transfusion Sanguine, Croix-Rouge Suisse, Centre Hospitalier Universitaire Vaudois, 1011 Lausanne, Switzerland.*

**Summary** Dose intensity may be an important determinant of the outcome in cancer chemotherapy, but is often limited by cumulative haematological toxicity. The availability of haematopoietic growth factors such as granulocyte colony-stimulating factor (G-CSF) and of peripheral blood progenitor cell (PBPC) transplantation has allowed the development of a new treatment strategy in which several courses of high-dose combination chemotherapy are administered for the treatment of solid tumours. PBPCs were mobilised before chemotherapy using 12 or 30 $\mu g\ kg^{-1}\ day^{-1}$ G-CSF (Filgrastim) for 10 days, and were collected by 2-5 leucaphereses. The yields of mononuclear cells, colony-forming units and CD34-positive cells were similar at the two dose levels of Filgrastim, and the numbers of PBPCs were sufficient for rescue following multiple cycles of chemotherapy. High-dose chemotherapy (cyclophosphamide 2.5 $g\ m^{-2}$ for 2 days, etoposide 300 $mg\ m^{-2}$ for 3 days and cisplatin 50 $mg\ m^{-2}$ for 3 days) was administered sequentially for a median of three cycles (range 1-4) to ten patients. Following the 30 evaluable cycles, the median duration of leucopenia $\leqslant 0.5 \times 10^9\ l^{-1}$ and $\leqslant 1.0 \times 10^9\ l^{-1}$ was 7 and 8 days respectively. The median time of thrombopenia $\leqslant 20 \times 10^9\ l^{-1}$ was 6 days. There was no cumulative haematological toxicity. The duration of leucopenia, but not of thrombopenia, was inversely related to the number of reinfused CFU-GM (granulocyte-macrophage colony-forming units). In the majority of patients, neurotoxicity and ototoxicity became dose limiting after three cycles of therapy. However, the average dose intensity delivered was about three times higher than in a standard regimen. The complete response rate in patients with small-cell lung cancers was 66% (95% CI 30-92%) and the median progression-free survival and overall survival were 13 months and 17 months respectively. These results are encouraging and should be compared, in a randomised fashion, with standard dose chemotherapy.

**Keywords:** small-cell lung cancer; intensive chemotherapy; peripheral blood progenitor cells; G-CSF

Small-cell lung cancer (SCLC) has been shown to be sensitive to a variety of single agents and combination chemotherapy, but the early emergence of resistant tumour cells remains the main cause of treatment failure. The effect of dose on tumour response is well established (Antman and Souhami, 1993), even if the impact on survival is unclear. There are suggestions that the initial administration of high doses of chemotherapy improves disease-free and overall survival in patients with limited disease (Arriagada *et al.*, 1993). It emphasises the importance of initial intensity in overcoming the development of resistant tumour cells. In experimental models, repeated cycles of chemotherapy have been shown, to be more cytotoxic than a single one (Teicher *et al.*, 1989). Based on these data, we attempted to develop an intensive regimen of combination chemotherapy that could be administered over multiple sequential cycles in unpretreated patients and to obtain a 3- to 4-fold intensification of chemotherapy compared with a standard regimen.

The chemotherapy regimen consisted of a combination of cyclophosphamide, etoposide and cisplatin. These agents were selected because of their dose-response in SCLC and their synergism in animal models (Johnson *et al.*, 1987). For each agent, a dose intensity which is known to produce only minimal non-haematological toxicity was chosen. Haematological toxicity was expected, but in order to reduce its duration and to avoid a cumulative exhaustion of haematopoiesis, peripheral blood progenitor cell (PBPC) transplantation and granulocyte colony-stimulating factor (Filgrastim) were used as haematological support. We planned to give four cycles of intensive combination chemotherapy, and according to dose intensity calculations (Hryniuk, 1988) the average relative dose intensity of our regimen was projected to be four times higher than a standard regimen administered for six cycles (Cortés Funes *et al.*, 1982). The feasibility of such an approach was the aim of our study.

Correspondence: S Leyvraz, Centre Pluridisciplinaire d'Oncologie, CHUV-BH 10, Rue du Bugnon 46, 1011 Lausanne, Switzerland
Received 19 October 1994; revised 13 February 1995; accepted 14 February 1995

### Patients and methods

Between June 1992 and January 1994, ten patients enrolled on the study. To be eligible, patients had to have normal cardiac, hepatic and renal functions, a performance status <2 according to ECOG (Oken *et al.*, 1982), to have given informed consent and to be <65 years old. Patients who had received chemotherapy or colony-stimulating factors in the preceding 12 months were excluded. The study was designed to include patients with a biopsy-proven SCLC, but one 30-year-old woman with metastatic breast cancer to the lung was also entered to test the feasibility of the programme. Acute toxicity was recorded according to the World Health Organization (1979) criteria. Limited disease was defined as that confined to one hemithorax, mediastinum and supraclavicular nodes, provided all volumes could be included in the same radiaotherapy field as the primary tumour. The presence of ipsilateral pleural effusion was classified as limited disease. All the other conditions were considered as extensive disease (Stahel *et al.*, 1989). Response was evaluated 4 weeks after the end of the high-dose chemotherapy regimen, by means of standard radiography, CT scan and bronchoscopy with biopsy. A complete response was defined as the complete disappearance of all clinical, radiological and, when applicable, pathological evidence of disease for a period of 4 weeks. Partial response was any response less than complete but with a greater than 50%

reduction in the sum of products of the cross-diameter of all measurable lesions. The study was approved by the ethical committee at our institution.

*Mobilisation and collection of peripheral blood progenitor cells*

Mobilisation of PBPCs was performed before chemotherapy using Filgrastim administered subcutaneously over 10 days at 12 $\mu g\ kg^{-1}\ day^{-1}$ (seven patients) and 30 $\mu g\ kg^{-1}\ day^{-1}$ (three patients). Starting on day 5, leucapheresis was performed a maximum of five times. In three patients, the harvesting procedure was carried out using a blood separator V-50 intermittent flow device (Haemonetics, Braintree, MA, USA) and in seven patients using a CS-3000 continuous flow device (Fenwal, Deerfield, IL, USA). The end points for each collection were either 4 h (Haemonetics) or 8 l of blood (Fenwal). Dimethylsulphoxide (DMSO 10% final concentration) was added to fractions containing mononuclear cells. The samples were frozen using a controlled rate freezer and stored in liquid nitrogen until use. CFU-GM (granulocyte-macrophage colony-forming units) were cultured using a double-layer semisolid agar technique as previously described (Pike and Robinson, 1970). Cells expressing the surface membrane CD34 antigen were identified with murine monoclonal antibodies QBEND10 (Immunotech, Marseille, France) or $4PCA_2$ (clone $8G_{12}$; Becton Dickinson, San Jose, CA, USA). At the time of reinfusion, PBPCs from one or two pheresis collections were rapidly thawed at 37°C and each administered over 10–20 min. A back-up bone marrow harvest was performed in the first four patients, but was never used.

*Treatment regimen*

The treatment programme consisted of cyclophosphamide 2.5 $g\ m^{-2}$ on days 1 and 2, etoposide 300 $mg\ m^{-2}$ on days 1, 2 and 3 and cisplatin 50 $mg\ m^{-2}$ on days 1, 2 and 3 and was planned to be repeated every 21 days for a total of four cycles. Mesna 4.0 $g\ m^{-2}\ day^{-1}$ was given as a continuous intravenous infusion on days 1 and 2, starting 1 h before cyclophosphamide. Hydration consisted of 4 l of 5% dextrose or normal saline supplemented with potassium chloride or magnesium sulphate. Frusemide 20 mg i.v. was given to maintain a urine output of $\geq$200 $ml\ h^{-1}$. Prophylactic antiemetic therapy consisted of ondansetron or granisetron with high-dose methylprednisolone. Lorazepam and metoclopramide were added if necessary. Ciprofloxacin and phenoxymethylpenicillin were given during the periods of neutropenia and broad-spectrum antibiotics were administered during febrile episodes. Platelet transfusions were administered for platelets counts lower than $10 \times 10^9\ l^{-1}$ or in case of haemorrhagic events. PBPCs were reinfused 24–48 h after the end of chemotherapy, followed by Filgrastim at 12 $\mu g\ kg^{-1}\ day^{-1}$ or 30 $\mu g\ kg^{-1}\ day^{-1}$ subcutaneously for 14 days or less in case of early recovery. Complete responders received 45 Gy radiotherapy at the primary tumour site and the mediastinum on completion of the treatment programme.

*Statistics*

Comparison between the groups was done using the Kolmogorov–Smirnov test (Siegel, 1956). Analysis of disease-free survival or overall survival was done using the Kaplan–Meier method (Kaplan and Meier, 1958). Dose intensity (DI) was calculated according to Hryniuk (1988) and Longo *et al.*, (1991).

**Results**

The patients had a median age of 50 years (range 30–62) and their median performance status was 1 (range 0–1). Except for the 30-year-old woman with metastatic breast cancer, five patients had limited (stage IIIa, two patients; stage IIIb, three patients) and four extensive SCLC. One patient with a severe superior vena cava syndrome received radiotherapy on the mediastinum (20 Gy) after PBPC collection, but before chemotherapy. One patient with SCLC had had a mastectomy for breast cancer 5 years previously and then received adjuvant chemotherapy and radiotherapy, as did the patient with breast cancer. Because of internal mammary and right supraclavicular lymph node recurrence, this last patient also had radiotherapy to the mediastinum 1 month before starting on the programme.

Thirty cycles were evaluable. Three patients completed four cycles, five received only three cycles and one patient had two cycles and another one cycle. The reason for not completing the four cycles was, in five patients, the severe non-haematological toxicity which was observed in the first three patients. One patient withdrew after two cycles and one after one cycle because of complications due to superior vena cava syndrome.

*PBPC mobilisation and collection*

After 5 days of Filgrastim, at the start of leucaphereses, the median leucocyte count was $67.7 \times 10^9\ l^{-1}$ (range 30.4–79.2). Following a median of four leucaphereses (range 2–5), the collections yielded medians of mononuclear cells, CFU-GM and CD34-positive cells of $12.9 \times 10^8\ kg^{-1}$ (range 4.2–20.8), $40.2 \times 10^4\ kg^{-1}$ (range 4.2–214.8) and $7.5 \times 10^6\ kg^{-1}$ (range 1.6–21.9) respectively and were not affected by the dose of Filgrastim.

*Toxicity*

The durations of leucopenia and thrombopenia were calculated from the day of PBPC reinfusion to recovery and as the number of days with low leucocyte or thrombocyte counts. The overall median time to leucocyte counts $\geq$0.5 and $\geq 1.0 \times 10^9\ l^{-1}$ was 9 (range 8–14) and 10 days (range 8–15) respectively. The median duration with a leucocyte count $\leq$0.5 and $\leq 1.0 \times 10^9\ l^{-1}$ was 7 days (range 5–12) and 8 days (range 6–13). As seen in Figure 1a, there was no cumulative neutropenia.

The median time to leucocyte count $\geq 1.0 \times 10^9\ l^{-1}$ for the first, second, third and fourth cycles of treatment was 10, 9, 10.5 and 10 days respectively. The median time to $\geq 1.0 \times 10^9\ l^{-1}$ leucocytes with either dose of Filgrastim was 10 days. Recovery from thrombocytopenia is demonstrated in Figure 1b with a median time to $\geq 20 \times 10^9\ l^{-1}$ of 11 days (range 9–15), without any cumulative thrombopenia.

The median number of CFU-GM $kg^{-1}$ reinfused after each cycle of chemotherapy was $8.32 \times 10^4\ kg^{-1}$ (range 1.69–61.0). There was a direct relationship between the number of reinfused CFU-GM and recovery of marrow function. When the number of CFU-GM $kg^{-1}$ was $\leq 10 \times 10^4\ kg^{-1}$, the median time to a leucocyte count of $\geq 1.0 \times 10^9\ l^{-1}$ was 11 days (range 9–15). It was 9 days (range 8–10) with a CFU-GM count of $>10 \times 10^4\ kg^{-1}$. This difference was highly significant ($P = 0.001$). The number of CFU-GM $kg^{-1}$ transfused had no influence on platelet recovery $\geq 20 \times 10^9\ l^{-1}$. After the administration of >10 or $\leq 10 \times 10^4\ kg^{-1}$ CFU-GM, median duration of platelet recovery was 10.5 days (range 9–14) and 12 days (range 9–15) respectively ($P>0.1$).

Proven infection developed during 37% of 30 cycles of treatments, with no correlation with Filgrastim dosage. There was also no correlation with the number of days on intravenous antibiotics, the length of stay in hospital and number of platelet or red cell transfusions. Duration of hospitalisation was the same during the first and subsequent cycles with a median of 18 days (range 15–44). The longest period of hospitalisation was observed in the patient with superior vena cava syndrome, who experienced severe oesophagitis and bacteraemia, possibly related to previous radiotherapy.

Neurotoxicity and ototoxicity were severe in the three patients who received four cycles of treatment, and this led

**Figure 1** (a) Median leucocyte count according to cycles. (b) median platelet count according to cycles.

us to cancel the administration of the fourth cycle in subsequent patients. The non-haematological toxicities are reported in Table I. Peripheral neuropathy WHO grade 1 occurred in two patients, grade 2 in four patients and grade 3 in one patient receiving two, three and four cycles of treatment respectively. Tinnitus occurred in three patients after one cycle of treatment. After three cycles of treatment, one patient developed moderate hearing loss, which also developed in the three patients receiving four cycles of treatment. Gastrointestinal toxicity, consisting of nausea and vomiting and mucositis and diarrhoea, was not dose-limiting. Only one patient had rectorrhagia following a transient ileus. Weight loss $\geq 10\%$ of the body mass occurred in three patients after two cycles, in five patients after three cycles and in all three patients after four cycles of treatment. All patients complained of fatigue, which imposed treatment delays in the majority of them.

### *Dose intensity*

Intensification of chemotherapy can be measured in three different ways: as dose per unit of time, as single dose intensity or as total dose delivery. Calculations and comparisons can be made based on planned treatment or on the actual delivered chemotherapy. We compared the planned doses of our regimen with a standard one in which the same three drugs were used (Cortès Funes *et al*., 1982). The single dose intensity and the total dose of cyclophosphamide were 8.3 and 5.5 times higher respectively, and of etoposide three and two times higher, and of cisplatin 1.9 and 1.25 times higher than the standard regimen. The planned average dose intensity per unit of time was increased 4.4-fold. However, owing to treatment delays and missing cycles, the actual delivered chemotherapy was a median of 1017 $mg\,m^{-2}\,week^{-1}$ (range 416–1360) of cyclophosphamide instead of the planned 1666 $mg\,m^{-2}\,week^{-1}$. It was 184 $mg\,m^{-2}\,week^{-1}$ (range 75–267) of etoposide instead of 300 $mg\,m^{-2}\,week^{-1}$ and 31 $mg\,m^{-2}\,week^{-1}$ (range 13–33) of

**Table I** Non-haematological toxicity (WHO grading)

| | Cycle number | | | |
|---|---|---|---|---|
| | *I* | *II* | *III* | *IV* |
| Peripheral neuropathy | 0 | 0 | 1 | 3 |
| Median (range) | | (0–1) | (0–2) | (2–3) |
| Hearing | 0 | 0 | 0 | 2 |
| Median (range) | (0–1) | (0–2) | (0–2) | |
| Nausea/vomiting | 2 | 2 | 2 | 2 |
| Median (range) | (1–3) | (0–3) | (1–3) | (1–2) |
| Mucositis | 0 | 0 | 0 | 0 |
| Median (range) | (0–1) | (0–1) | (0–3) | |
| Diarrhoea | 1 | 1 | 1 | 1 |
| Median (range) | (0–2) | (0–4 | (0–2) | |
| Weight loss | 1 | 1 | 2 | 2 |
| Median (range) | (0–1) | (0–2) | (0–2) | |

cisplatin instead of 50 $mg\,m^{-2}\,week^{-1}$. The actual average percentage of dose intensity for all three drugs was 61% of the planned dose intensity. Even if data on actual dose delivery of the standard regimen are not available, we estimate that the escalation in dose intensity of our regimen was about 3-fold.

### *Anti-tumour activity*

Response evaluation was not the primary aim of this study. The patient with breast cancer and lung metastases developed a complete clinical remission, lasting 24 + months. Among the nine patients with SCLC, six were complete responders confirmed by bronchoscopy and three partial responders. With a median follow-up period of 20 months, progression was detected in six of the nine responding patients; of these, four had recurrences at the primary site and four had distant metastases (pleura two patients; liver, three patients; and brain, two patients). The median progression-free survival and overall survival were 13 and 17 months respectively.

### Discussion

High-dose chemotherapy has commonly been administered as a single cycle of high peak intensity and resulted in higher cure rates in patients with acute leukaemia or lymphoma and encouraging results in some solid tumours (Antman and Souhami, 1993). To improve the management of SCLC, it is necessary to develop a treatment capable of circumventing or minimising the impact of drug resistance. The intensification of chemotherapy appears to be a potentially effective treatment strategy. However, the majority of the studies focused on single dose intensity instead of total dose or dose rate intensity. For long-term remissions and cure of chemotherapy-sensitive malignancies, multiple cycles of standard combination chemotherapy are required. The same may be true for high-dose chemotherapy in the treatment of less responsive solid tumours. The availability of haematopoietic growth factors and peripheral blood progenitor cells allowed us to consider the possibility of developing a treatment approach in which repeated courses of high-dose combination chemotherapy were administered sequentially over four cycles with an increased projected average dose intensity of more than four times that of a standard regimen. However, the non-haematological toxicity due to cisplatin became the dose-limiting toxicity (Leyvraz *et al*., 1993). All patients receiving four cycles of therapy experienced severe neuro- and ototoxicity which prevented the administration of the fourth cycle in subsequent patients. Altogether, missing cycles and treatment delays led to the chemotherapy actually delivered being 61% of the planned dose intensity. This remained, nevertheless, about three times the intensity of a standard regimen. In comparison, the increase in dose intensity of accelerated weekly schedules is only 1.5 times that of conventional three-weekly regimens (Ardizzoni *et al*., 1993)

and the addition of G-CSF does not significantly affect further intensification (Miles et al., 1994).

The collection of PBPCs before chemotherapy allowed easy planning of the sequential intensive treatment. In the few reports of multiple cycles of high-dose chemotherapy, the PBPCs were collected at the end of each cycle of chemotherapy at the time of mononuclear cell and platelet recovery, making the timing of leucapheresis less predictable (Crown et al., 1992; Tepler et al., 1993) and possibly exhausting the ability to mobilise PBPCs and the capacity of stem cells to reconstitute marrow functions (Shea et al., 1994). The optimum method for mobilisation of PBPCs is still open to question, even if there are suggestions that chemotherapy, followed by colony-stimulating factors, induces a higher yield than any agent alone (Teshima et al., 1993).

Filgrastim was administered after the reinfusion of PBPCs. When PBPCs or haematopoietic growth factors are used alone after high-dose chemotherapy, haematological recovery time is prolonged compared with the administration of both modalities (Kritz et al., 1992; Shimazaki et al., 1993; van der Wall et al., 1994). The dose of Filgrastim did not affect the rate of leucocyte or platelet recovery or modify the incidence of infections, the number of days on antibiotics or the duration of hospitalisation. Low-dose Filgrastim might be as effective. Indeed, in a randomised trial, even though the dose of 20 $\mu g\ kg^{-1}\ day^{-1}$ G-CSF was marginally more effective than 5 or 10 $\mu g\ kg^{-1}\ day^{-1}$ following bone marrow transplantation, the dose of 5 $\mu g\ kg^{-1}\ day^{-1}$ was recommended for further trials (Linch et al., 1993).

The anti-tumour activity of our regimen was substantial, with a 66% complete response rate (95% CI 30–92%), which is similar to other intensive treatment strategies (Souhami et al., 1985; Johnson et al., 1987). It resulted in a median progression-free survival of 13 months and an overall survival of 17 months, possibly because of the selection of patients with good physical condition. However, these results are encouraging and in the range of the best reported results for the treatment of patients with limited disease (Elias et al., 1993).

Further improvements in the design of intensive treatment strategies may be possible. Radiotherapy might have a role in controlling bulky or localised disease and should be integrated without compromising the delivery of chemotherapy. Other combinations of chemotherapeutic agents might be more suitable for sequential intensive treatment. The intensification of our regimen was mainly due to the ability to increase the dose intensity of cyclophosphamide eight times and that of etoposide 1.8 times. Cisplatin dosage could not be increased significantly and was the cause of limiting toxicity. A combination of alkylating agents or the use of anthracyclines may be more appropriate (Murray, 1987). We are currently testing the sequential administration of multiple cycles of high-dose ICE (ifosfamide–carboplatin–etoposide), which will be evaluated in a randomised fashion against conventional dose therapy.

**Acknowledgements**
We thank Dr S Pampallona for his advice in statistical analysis and Professor M Glauser and Dr M Cometta from the Infectious Disease Department for their help in caring for these patients. We thank Ms M Gonin for secretarial assistance.

### References

ANTMAN KH AND SOUHAMI RL. (1993). High-dose chemotherapy in solid tumours. *Ann. Oncol.*, **4**, (Suppl. 1), S29–S44.

ARDIZZONI A, VENTURINI M, CRINO L, SERTOLI MR, BRUZZI P, PENNUCCI MC, MARIANI GL, GARRONE O, BRACARDA S, ROSSO R AND VAN ZANDWIJK N. (1993). High dose-intensity chemotherapy, with accelerated cyclophosphamide–doxorubicin etoposide and granulocyte–macrophage colony stimulating factor, in the treatment of small cell lung cancer. *Eur. J. Cancer*, **29**, 687–692.

ARRIAGADA R, LE CHEVALLIER T, PIGNON JP, RIVIERE A, MONNET I, CHOMY P, TUCHAIS C, TARAYE M AND RUFFIE P. (1993). Initial chemotherapeutic doses and survival in patients with limited small-cell lung cancer. *N. Engl. J. Med.*, **329**, 1848–1852.

CORTES FUNES H, DOMINGUEZ P, PEREZ TORRUBIA A, LANZOS E, MENDES M AND MENDIOLA C. (1982). Treatment of small cell lung cancer with a combination of V16-213 and cyclophosphamide with cisplatin or radiotherapy. *Cancer Chemother. Pharmacol.*, **7**, 181–186.

CROWN J, WASSHERHEIT C, HAKES T, FENNELLY D, REICH L, MOORE M, CURTIN J, RUBIN SC, REICHMAN B, YAO TJ, GILEWSKI T, GULATI S, MARKMAN M AND NORTON L. (1992). Rapid delivery of multiple high-dose chemotherapy courses with granulocyte colony-stimulating factor and peripheral blood-derived haematopoietic progenitor cells. *J. Natl Cancer Inst.*, **84**, 1935–1936.

ELIAS AD, AYASH L, FREI III E, SKARIN AT, HUNT M, WHEELER C, SCHWARTZ G, MAZANET R, TEPLER I, EDER JP, McCAULEY M, HERMAN T, SCHNIPPER L AND ANTMAN K. (1993). Intensive combined modality therapy for limited-stage small-cell lung cancer. *J. Natl Cancer Inst.*, **85**, 559–566.

HRYNIUK W. (1988). The importance of dose intensity in outcome of chemotherapy. In *Important Advances in Oncology*, De Vita Jr VT, Hellman S and Rosenberg SA.(eds) pp. 121–141. JB Lippincott: Philadelphia.

JOHNSON D, DE LEO MJ, HANDE KR, WOLFF SN, HAINSWORTH JD AND GRECO FA. (1987). High dose induction chemotherapy with cyclophosphamide, etoposide and cisplatin for extensive small-cell lung cancer. *J. Clin. Oncol.*, **5**, 703–709.

KAPLAN EL AND MEIER P. (1958). Nonparametric estimation from incomplete observations. *J. Am. Stat. Assoc.*, **53**, 457–481.

KRITZ A, CROWN JP, MOTZER RJ, REICH LM, HELLER G, MOORE MP, HAMILTON N, YAO TJ, HEELAN RT, SCHNEIDER JG, MOORE MAS, McCORMICK B, GILEWSKI TA, O'REILLY RJ, GULATI SC AND NORTON L. (1992). *Cancer*, **71**, 2515–2521.

LEYVRAZ S, KETTERER N, VUICHARD P, VON FLIEDNER V, LEJEUNE F, SCHNEIDER P, GROB JP AND BACHMANN F. (1993). Sequential high-dose combination chemotherapy with granulocyte colony-stimulating factor and peripheral blood progenitor cells in patients with solid tumours: intensification limited by nonhaematologic toxic effects. *J. Natl Cancer Inst.*, **85**, 1962–1964.

LINCH DC, SCARFFE H, PROCTOR S, CHOPRA R, TAYLOR PRA, MORGENSTERN G, CUNNINGHAM D, BURNETT AK, COWLEY JC, FRANKLIN IM, BELL AJ, LISTER TA, MARCUS RE, NEWLAND AC, PARKER AC AND YVER A. (1993). Randomized vehicle-controlled dose-finding study of glycosylated recombinant human granulocyte colony-stimulating factor after bone marrow transplantation. *Bone Marrow Transplantation*, **11**, 307–311.

LONGO DL, DUFFEY PL, DeVITA JR VT, WESLEY MN, HUBBARD SM AND YOUNG RC. (1991). The calculation of actual or received dose intensity: a comparison of published methods. *J. Clin. Oncol.*, **9**, 2042–2051.

MILES DW, FOGARTY O, ASH CM, RUDD RM, TRASK CWL, SPIRO SG, GREGORY WM, LEDERMANN JA, SOUHAMI RL AND HARPER PG. (1994). Received dose-intensity: a randomized trial of weekly chemotherapy with and without granulocyte colony-stimulating factor in small-cell lung cancer. *J. Clin. Oncoal.*, **12**, 77–82.

MURRAY N. (1987). The importance of dose and dose intensity in lung cancer chemotherapy. *Semin. Oncol.*, **14**, (Suppl. 4), 20–28.

OKEN MM, CREECH RH, TORMEY DC, HORTON J, DAVIS TE, McFADDEN ET AND CARBONE PP. (1982). Toxicity and response criteria of the Eastern Cooperation Oncology Group. *Am. J. Clin. Oncol.*, **5**, 649–655.

PIKE BL AND ROBINSON WA. (1970). Human bone marrow colony growth in agar-gel. *Cell Physiol.*, **76**, 77–84.

SHEA TC, MASON JR, BRESLIN M, BISSEN E, MULLER M AND TAETLE R. (1994). Reinfusion and serial measurements of carboplatin-mobilized peripheral blood progenitor cells in patients receiving multiple cycles of high-dose chemotherapy. *J. Clin. Oncol.*, **12**, 1012–1020.

SHIMAZAKI C, OKU N, UCHIYAMA H, YAMAGATA N, TATSUMI T, HIRATA T, ASHIHARA E, OKAWA K, GOTO H, INABA T, FUJITA N, HARUYAMA H AND NAKAGAWA M. (1994). Effect of granulocyte colony-stimulating factor on hematopoietic recovery after peripheral blood progenitor cell transplantation. *Bone Marrow Transplant*, **13**, 271–275.

SIEGEL S. (1956). *Non-parametric Statistics*, pp. 127–136. McGraw Hill: London.

SOUHAMI RL, FINN G, GREGORY WM, BIRKHEAD BG, BUCKMAN R, EDWARDS D, GOLDSTONE AH, HARPER PG, SPIRO SG, TOBIAS JS AND GEDDES D. (1985). High-dose cyclophosphamide in small-cell carcinoma of the lung. *J. Clin. Oncol.*, **3**, 958-963.

STAHEL RA, GINSBERG R, HAVEMANN K, HIRSCH FL, IHDE DC, JASSEM J, KARRER K, MAURER LH, OSTERLIND K AND VAN HOUTTE P. (1989). Staging and prognostic factors in small cell lung cancer: a consensus report. *Lung Cancer*, **5**, 119-126.

TEICHER BA, HOLDEN SA, EDER JP, BRANN TW, JONES SM AND FREI III E. (1989). Influence of schedule on alkylating agent cytotoxicity in vitro and in vivo. *Cancer Res.*, **49**, 5994-5998.

TEPLER I, CANNISTRA SA, FREI III E, GONIN R, ANDERSON KC, DEMETRI G, NILHOFF J, GOODMAN H, MUNTZ H, MUTO M, SHEETS E, ELIAS AD, MAZANET R, WHEELER C, AYASH L, SCHWARTZ G, McCAULEY M, GAYNES L, HARVEY S, SCHNIPPER LE AND ANTMAN K. (1993). Use of peripheral-blood progenitor cells abrogates the myelotoxicity of repetitive outpatient high-dose carboplatin and cyclophosphamide chemotherapy. *J. Clin. Oncol.*, **11**, 1583-1591.

TESHIMA T, HARADA M, TAKAMATSU Y, MAKINO K, INABA S, AKASHI K, KONDO S, TANAKA T, ISHII E AND NIHO Y. (1993). Granulocyte colony-stimulating factor (G-CSF)-induced mobilization of circulating haematopoietic stem cells. *Br. J. Haemat.*, **84**, 570-573.

VAN DER WALL E, RICHEL DJ, HOLTKAMP MJ, SLAPER-CORTENBACH ICM, VAN DER SCHOOT CE, DALESIO O, NOOIJEN WJ, SCHORNAGEL JH AND RODENHUIS S. (1994). Bone marrow reconstitution after high-dose chemotherapy and autologous peripheral blood progenitor cell transplantation: effect of graft size. *Ann. Oncol.*, **5**, 795-802.

WORLD HEALTH ORGANIZATION. (1979). *WHO Handbook for Reporting the Results of Cancer Treatment*, Offset publication No. 48. WHO: Geneva.